# Morphological, genetic and ecological divergence in near-cryptic bryophyte species widespread in the Holarctic: the *Dicranum acutifolium* complex (Dicranales) revisited in the Alps

**DOI:** 10.1007/s10265-024-01534-3

**Published:** 2024-03-23

**Authors:** Thomas Kiebacher, Péter Szövényi

**Affiliations:** 1 Department of Botany, Stuttgart State Museum of Natural History, Rosenstein 1, 70191 Stuttgart, Germany; 2 Department of Systematic and Evolutionary Botany, University of Zurich UZH, Zollikerstrasse 107, 8008 Zurich, Switzerland; 3 Zurich-Basel Plant Science Center (PSC), ETH Zürich, Tannenstrasse 1, 8092 Zurich, Switzerland

**Keywords:** Adaptive divergence, Ecological vicariance, Incomplete lineage sorting, Introgression, Substrate specificity

## Abstract

There is mounting evidence that reproductively isolated, but morphologically weakly differentiated species (so-called cryptic species) represent a substantial part of biological diversity, especially in bryophytes. We assessed the evolutionary history and ecological differentiation of a species pair, *Dicranum brevifolium* and *D. septentrionale*, which have overlapping ranges in the Holarctic. Despite their morphological similarity, we found similar genetic differentiation as between morphologically well-differentiated *Dicranum* species. Moreover, we detected gene tree discordance between plastid and nuclear markers, but neither of the two datasets resolved the two as sister species. The signal in *trnL–trnF* better reflects the morphological and ecological affinities and indicates a close relationship while ITS sequence data resolved the two taxa as phylogenetically distantly related. The discordance is probably unrelated to the ecological differentiation of *D. septentrionale* to colonise subneutral to alkaline substrates (vs. acidic in *D. brevifolium*), because this ability is rare in the genus and shared with *D. acutifolium*. This taxon is the closest relative of *D. septentrionale* according to the *trnL–trnF* data and does not share the discordance in ITS. We furthermore demonstrate that beside *D. acutifolium*, both *D. septentrionale* and *D. brevifolium* occur in the Alps but *D. brevifolium* is most likely rarer. Based on morphological analyses including factor analysis for mixed data of 45 traits we suggest treating the latter two as near-cryptic species and we recommend verifying morphological determinations molecularly.

**Supplementary Information:**

The online version contains supplementary material available at 10.1007/s10265-024-01534-3.

## Introduction

The application of molecular methods has opened a completely new perception of biological diversity on Earth. In bryophytes, the second largest group of land plants after vascular plants, it has led to the discovery of substantial species diversity in many lineages that had not been recognised in earlier morphological concepts (e.g., Bakalin et al. 2020; Fernandez et al. 2006; Hedenäs and Eldenäs 2007; see Renner 2020 for a review). Such taxa, where molecular data indicate reproductive isolation without substantial morphological differentiation, are usually referred to as cryptic. Most commonly, cryptic taxa are phylogenetically closely related and often reflect differentiation along geographical or ecological gradients. The lack of morphological differentiation can then be explained by a recent origin form a common ancestor without substantial phenotypic change (Struck et al. 2018). By contrast, examples of more deeply divergent cryptic species are rare and can result from morphological stasis or convergence (Renner et al. 2013; Struck et al. 2018).

Here we assess an example of two deeply divergent species in the *Dicranum acutifolium* complex: *Dicranum brevifolium* (Lindb.) Lindb. and *D. septentrionale* Tubanova & Ignatova. The latter species was first reported through variation in sequences of the nuclear ITS region analysing accessions of *D. brevifolium* from Russia (Tubanova et al. 2010). Based on the molecular clustering of specimens, minor morphological differences were found between the two species; i.e., predominantly elongated distal and median lamina cells in *D. septentrionale* (vs. isodiametric in *D. brevifolium*) and usually longer basal cells. Subsequently, Lang et al. (2014) also considering specimens from Scandinavia, performed a cluster analysis of 34 morphological characters that failed to unambiguously discern the two species while the phylogenetic analysis of the concatenated dataset of the nuclear ITS region and five plastid loci indicated that the two taxa are not closely related (Lang et al. 2015). *Dicranum septentrionale* was resolved as falling outside of the clade containing the morphologically similar species *D. brevifolium* and *D. acutifolium* (Lindb. & Arnell) C.E.O.Jensen. These latter two species appeared to be genetically more closely related to species which differ strongly morphologically, e.g., *D. angustum* Lindb. and *D. bonjeanii* De Not.

**Fig. 1:**
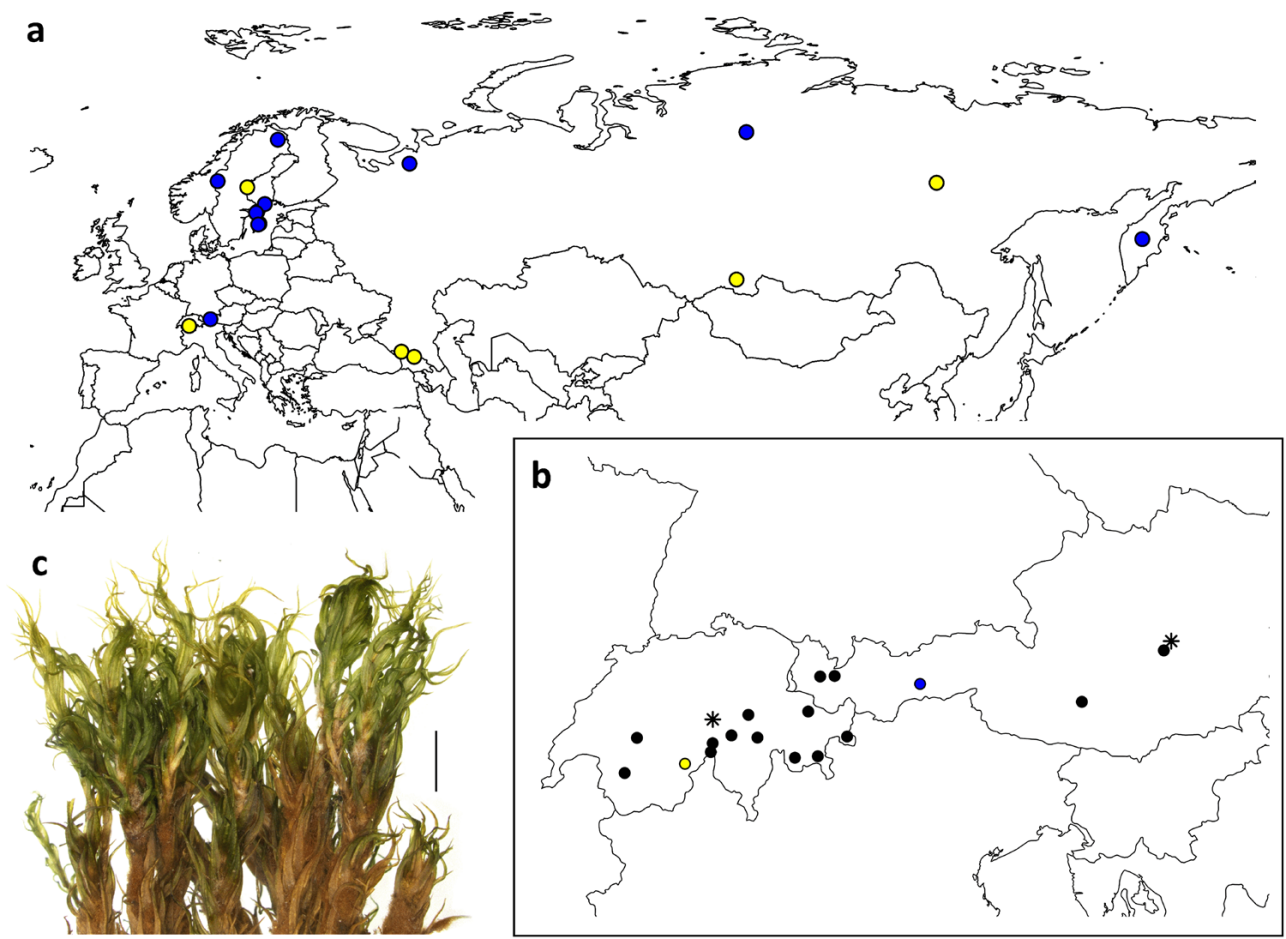
**a** Distribution of *Dicranum brevifolium* (yellow dots) and *D. septentrionale* (blue) in Eurasia according to Tubanova et al. (2010) and Lang et al. (2014) considering only molecularly analysed specimens. **b** Sampling of *D. brevifolium* s.l. (black dots) and *D. acutifolium* (asterisks) in the Alps. **c** Plant of *Dicranum brevifolium* s.l. in dry state, scale bare = 2 mm

To date, no clear geographical pattern has been documented that could explain the genetic differentiation of *D. brevifolium* and *D. septentrionale*, both of which are known from across Eurasia (Lang et al. 2014; Tubanova et al. 2010; Fig. 1). Therefore, the genetic differentiation of the two species may coincide with ecological differences. Both are known to inhabit mountainous regions, and less commonly, lowland areas (Lang et al. 2014; Tubanova et al. 2010). However, they may occur on divergent substrates, but to date, this hypothesis has not been investigated. Different substrate affinities have often been observed between closely related plant species, including bryophytes (Kiebacher et al. 2022).

In Central Europe, taxonomic assessment of the *D. acutifolium* complex is incomplete and ambiguous. The complex comprises the morphologically similar species *D. acutifolium*, *D. bardunovii* Tubanova & Ignatova., *D. brevifolium* and *D. septentrionale* (Lang et al. 2014). *Dicranum brevifolium* (sub *D. muehlenbeckii* subsp. *brevifolium* (Lindb.) J.J. Amann or *D. muehlenbeckii* var. *brevifolium* Lindb.) was mentioned from Switzerland by Limpricht (1886) and Amann et al. (1918) and by Breidler (1891) from Austria, but all authors claimed morphological differences to Nordic collections. Certainly, these records include *D. acutifolium* and *D. septentrionale*. Until recently, reliable records for *D. acutifolium* from the Alps were very sparse and its occurrence was not accepted in Grims (1999) for Austria and in Meinunger and Schröder (2007) for Germany. Only recently Hedenäs and Bisang (2004) or Schlüsslmayr (2019) seriously reported its presence in the Eastern Alps and so far, only one Central European sample of the complex from Switzerland (belonging to *D. brevifolium*) and one from Austria (belonging to *D. septentrionale*) have been molecularly examined (Lang et al. 2014).

By analysing multiple accessions of *D. acutifolium* and *D. brevifolium* s.l. (*D. brevifolium* + *D. septentrionale*) from different substrates in the Alps molecularly and morphologically and including available sequence data from previous work in the phylogenetic analyses we address the discordance between the phylogenetic and morphological signal in *D. brevifolium* and *D. septentrionale*.

Specifically, (i) we test the consistency of the phylogenetic signal in nuclear and plastid sequence data and (ii) if specimens from the Alps can be assigned to one of the two species based on morphological characteristics. Furthermore, (iii) we explore whether the two taxa differ regarding substrate specificity and (iv) we provide information about their occurrence in the Alps.

## Material and methods

### Taxon sampling and morphological analyses

To provide specimens < 20 years old (suitable for molecular examination) we sent requests to GJO, W and Z + ZT and contacted seven private collectors active in the Alps (see acknowledgements) who we expected to hold collections of the complex. In this way we succeeded in providing 15 specimens from the Austrian (4 specimens) and Swiss (11) Alps which according to an initial morphological examination were assigned to *D. brevifolium* s.l. Furthermore, we considered two specimens of *D. acutifolium* (Fig. 1). To examine the specimens of *D. brevifolium* s.l. morphologically we applied two approaches. (a) We asked an experienced bryologist to determine the specimens using the distinguishing characters presented by Tubanova et al. (2010) and Lang et al. (2014) prioritising the shape of the median and upper lamina cells and the length of the basal cells as pointed out by Tubanova et al. (2010). In case of overlapping characteristics or discordance between the characters, the determinator had the option to assign multiple taxon names, starting with the best fit taxon name. (b) We compiled a data set of 45 morphological traits comprising the characters presented by Tubanova et al. (2010) and Lang et al. (2014) and further characters commonly used for species distinction in the genus (see e.g., Hedenäs and Bisang 2004) and analysed it using multivariate statistics. (i) We measured length and width of 5 leaves of one well-grown shoot of each specimen and estimated distal dorsal costa and upper leaf margin ornamentation and the shape of the base of these leaves. (ii) We measured length and width of 10 basal cells of each of the 5 leaves and counted the number of pores per cells. And (iii), we visually estimated the portion of elongate, isodiametric, oblate and triangular lamina cells at ½–$${\raise0.5ex\hbox{$\scriptstyle 1$} \kern-0.1em/\kern-0.15em \lower0.25ex\hbox{$\scriptstyle 3$}}$$ above the leaf base. We calculated minimum, maximum and average values, as well as leaf and basal cell length/width ratio per specimen to compile the spreadsheet of traits and analysed it using factor analysis for mixed data (FAMD; Pagès 2004) with the FAMD function in the R package FactoMineR (Le et al. 2008). The technique combines principal component and multiple correspondence analyses and allows to simultaneously analyse quantitative and qualitative variables. The spreadsheet including a more detailed description how the traits were estimated is available in Supplementary Information 1. We ran the analysis in R version 4.1.3 (R Core Team 2022) and summarized results graphically using the plotly package (Sievert 2020). Furthermore, we tested for species specific differences individually for each trait using the Wilcoxon-signed-rank test implemented in the ggpubr package (Kassambara 2023).

### Marker selection

To identify plastid markers that are sufficiently variable to resolve the species of the *D. acutifolium* complex, we used the dataset of Lang et al. (2015) and generated phylogenetic trees independently for each of the five loci examined (*rpoB*, *rps19–rpl2*, *psbA–trnH*, *trnL–trnF*, *trnT–rps4*). For simplicity, we reduced the set of accessions to all representatives of *D. brevifolium*, *D. acutifolium* and *D. septentrionale* and at least two (if available) representatives of the other species and *Holomitrium arboreum* Mitten as an outgroup species. We aligned the sequences using the AlignSeqs function of the DECIPHER package (Wright 2015) with default settings in R version 4.1.3 (R Core Team 2022) and subsequent manual corrections. We scored indel data using the simple coding method (Simmons and Ochoterena 2000) and ran Bayesian Inference (BI) analyses using MrBayes v.3.2.7a (Ronquist et al. 2012). We specified a GTR + G + I model, a sample frequency of 100, stop rule set to yes with critical value for the topological convergence diagnostic set to 0.01 and default settings for all other parameters. Only *trnL–trnF* differentiated *D. brevifolium*, *D. acutifolium* and *D. septentrionale* from each other and from other species (Figs. S1−5 in Supplementary Information 2), thus we proceeded to sequence this locus and the nuclear ITS region. We a priori selected the latter because it had more parsimony informative sites than the five plastid markers combined (Lang et al. 2015).

### DNA extraction and sequencing

The protocols to generate the sequence data followed previous work (Kiebacher et al. 2021, 2022). In brief, genomic DNA was extracted using the NaOH method (Werner et al. 2002), we targeted the nuclear ITS region including partial sequence of 18S rRNA gene, ITS1, 5.8S rRNA gene, ITS2 and partial sequence of 26S rRNA gene using the primers m-18-S (Spagnuolo et al. 1999) and m-25-R (Stech and Frahm 1999), and the plastid spacer *trnL–trnF* including partial sequences of the *trnL*- and *trnF*-genes using the primers TabC and TabF (Taberlet et al. 1991). Reagents and volumes for the PCR reaction were the same as is described in Kiebacher et al. (2021), except that we used a 2.5 µg/ml solution of bovine serum albumin instead of water. Cycling for ITS followed Kiebacher et al. (2021) and for the *trnL–trnF* locus it started with 3-min initialisation at 94 °C, followed by 40 cycles of 1 min at 94 °C, 1 min at 49 °C, and 1 min at 72 °C, and a final extension step of 5 min at 72 °C. Purification of the PCR product, sequencing and editing of raw sequences were again performed following Kiebacher et al. (2021). Amplification of both loci failed for three of the specimens of *D. brevifolium* s.l. which we excluded from the subsequent analyses. For two specimens of *D. brevifolium* s.l. and one of *D. acutifolium*, only ITS sequences could be generated.

### Molecular data set and analyses

For the phylogenetic analyses we considered the set of accessions used for marker selection and extended it with accessions retrieved from Genbank. Specifically, we included accessions of *D. bardunovii*, *D. howellii* Renauld & Cardot*, D. ignatovii* Tubanova & Fedosov*, D. japonicum* Mitten*, D. laevidens* R.S.Williams and *D. muehlenbeckii* Bruch & Schimp. which were identified as similar to ITS or *trnL–trnF* sequences of our accessions of *D. brevifolium* s.l. based on BLAST searches using the megablast algorithm (https://blast.ncbi.nlm.nih.gov/Blast.cgi) with default settings. From this raw set of accessions, we excluded sequences with low coverage (less than 70% of the target locus) which applied to one *trnL–trnF* (GenBank accession number DQ462591) and one ITS sequence of *D. septentrionale* (HQ830340). The final dataset comprised 31 species of *Dicranum*, each represented by at least two accessions, and the outgroup taxon *Chorisodontium aciphyllum* (Hook.f. & Wilson) Broth. *Dicranum brevifolium* s.l. was represented by the 14 accessions from the Alps (two of which were from previous studies) and five accessions of *D. brevifolium* and nine of *D. septentrionale* from other regions. We aligned *trnL–trnF* sequences using the same function and software as used for marker selection and ITS sequences using the online interface of MAFFT v7 (Katoh and Standley 2013) applying the E-INS-i strategy, default settings for all other parameters and subsequent manual corrections. We scored indels for both alignments using simple coding (Simmons and Ochoterena 2000) and performed BI and Maximum Likelihood (ML) analyses. Bayesian Inference was performed with the same software and settings as for marker selection and convergence of the runs was checked using Tracer v1.7.2 (Rambaut et al. 2018). For ML analyses we used RAxML v8.2.4 (Stamatakis 2014), also specified the GTR + G + I model and stopped bootstrap analysis automatically using the autoMRE command. Support for the nodes of the best scoring tree out of 50 independent ML runs was assessed using the thorough bootstrapping algorithm with the extended majority rule bootstopping criterion. We summarised the support of nodes from the two approaches using TreeGraph 2 (Stöver and Müller 2010) displaying the BI topology.

### Substrate reaction

To classify the substrate of specimens of *D. brevifolium* s.l., including specimens for which sequence data were generated in previous studies, we defined two classes of substrate reaction, acidic (A) and subneutral to alkaline (N). To assign specimens to either of the two we used the bedrock type at the collection site and anticipated that substantial content of carbonate results in subneutral to alkaline conditions and that low or no content of carbonate results in acidic conditions. Bedrock type was derived from label information, geological maps, field observations of the collectors, and the evaluation of soil reaction affinities of associated species. For Switzerland, we used the geological map GeoCover of the Swiss Federal Office of Topography Swisstopo (available online; www.swisstopo.admin.ch) which combines information from different sources listed in Swisstopo (2021). For Austria, we used Rockenschaub and Nowotny (2009) and for Sweden we used the online bedrock map provided by SGU Geological Survey of Sweden (https://apps.sgu.se/kartvisare/kartvisare-berg-50-250-tusen.html). We succeeded in classifying 23 of the 29 specimens of *D. brevifolium* s.l. considered. For the remaining specimens, information on bedrock type was not available from labels and neither the locality nor the geological maps were accurate enough to reliably classify them. For other *Dicranum* species, we classified the substrate reaction requirements according to our expertise and indications in the literature (Chien et al. 1999; Dierssen 2001; Hill et al. 2007; Ireland 2007; Urmi 2010; van Zuijlen et al. 2023), which required an additional intermediate class for species that occur on moderately acidic to subneutral substrates.

## Results

### Molecular and morphological species assignment

The *trnL–trnF* alignment comprised 471 positions, of which 48 were variable and 47 were parsimony-informative. The alignment of the nrITS region comprised 883 positions, 122 were variable and 91 were parsimony-informative.

**Fig. 2:**
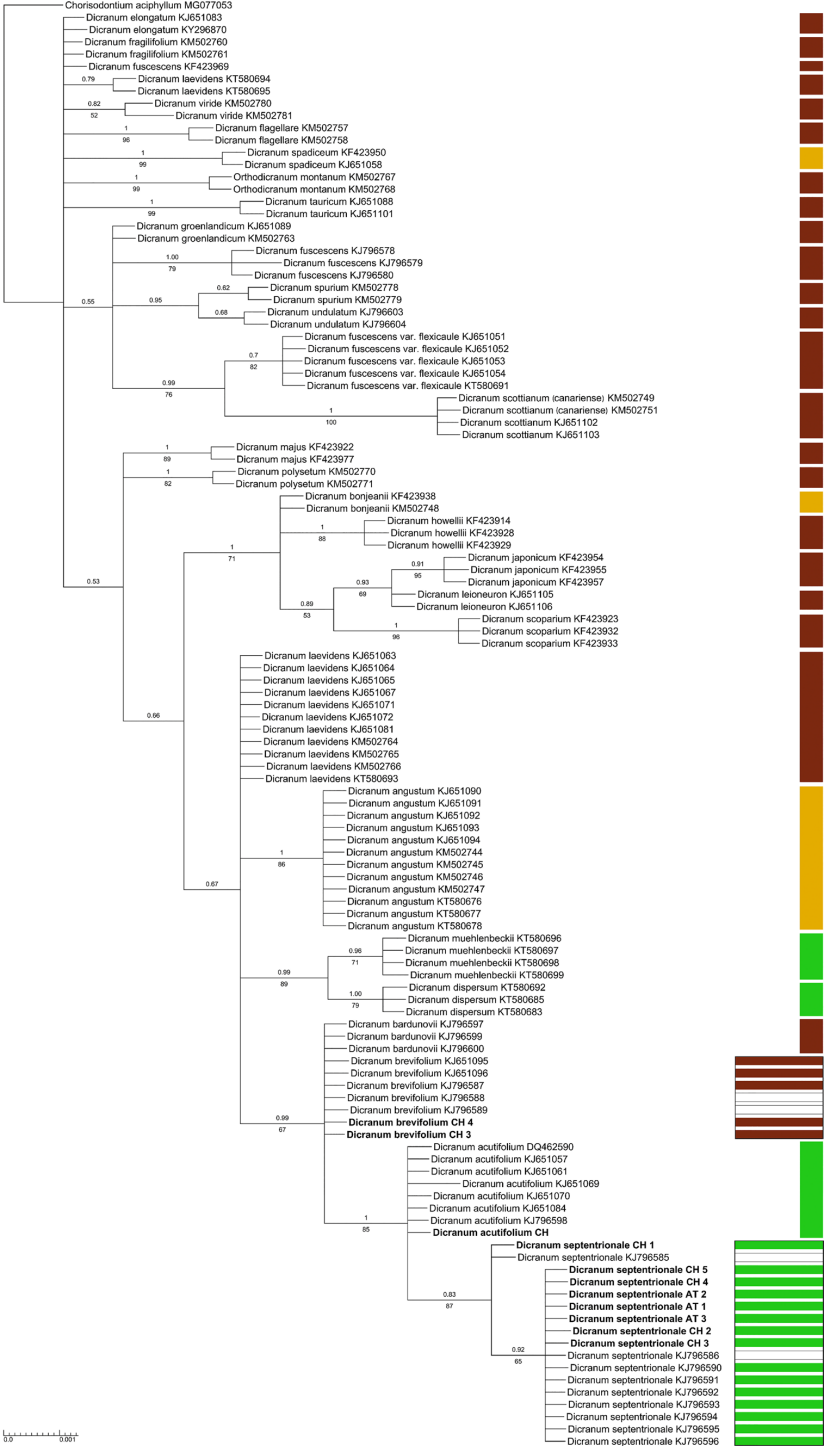
Bayesian inference from plastid *trnL*–*trnF* sequence data. Numbers above branches are Bayesian posterior probabilities ≥ 0.5, numbers below branches are bootstrap support values ≥ 50 of branches obtained from maximum likelihood analysis of the same dataset. New accessions are in bold, accessions retrieved from GenBank are followed by the accession number. For voucher information see Table 1. Colours indicate substrate reaction requirements of species and for *Dicranum brevifolium* and *D. septentrionale* the substrate reaction at the collection sites of each specimen: brown, acidic; ochre, moderately acidic to subneutral; green, subneutral to alkaline; blank fields, unknown

**Fig. 3:**
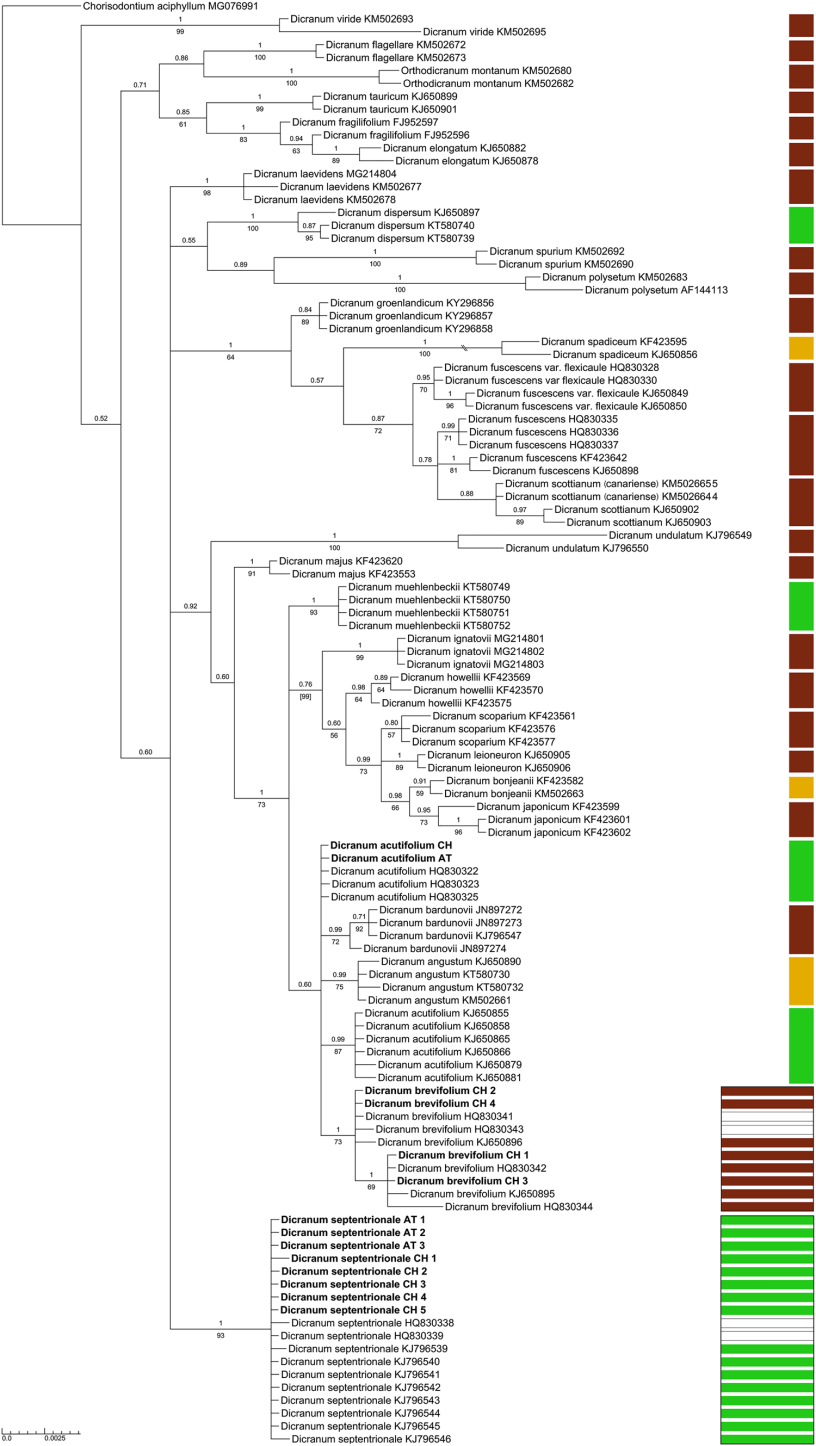
Bayesian inference from nuclear ITS sequence data. Numbers above branches are Bayesian posterior probabilities ≥ 0.5, numbers below branches are bootstrap support values ≥ 50 of branches obtained from maximum likelihood analysis of the same dataset. New accessions are in bold, accessions retrieved from GenBank are followed by the accession number. For voucher information see Table 1, for colour coding see Fig. 2

In agreement with the morphological diagnosis the phylogenetic analysis of ITS data resolved the two accessions of *D. acutifolium* among accessions of this species examined in earlier studies (Fig. 2). Of the 12 Alpine accessions of *D. brevifolium* s.l. for which we could generate sequences, four were resolved among accessions of *D. brevifolium* and eight among accessions of *D. septentrionale* (Table 1, Fig. 2). This molecular species assignment was consistent with the assignment in *trnL–trnF* except for one accession of *D. acutifolium* and two of *D. brevifolium* for which *trnL–trnF* sequences could not be generated (Table 1, Fig. 3). In the morphological determination, four accessions of *D. brevifolium* s.l. were assigned to *D. brevifolium*, four to *D. septentrionale* and for the four others, the morphological affinities were ambiguous (Table 1). The unambiguous determinations matched the molecular assignment in no more than two cases for *D. septentrionale* (Table 1). The four specimens with ambiguous morphological affinities were molecularly either resolved among accessions of *D. brevifolium* (2 specimens) or *D. septentrionale* (2 specimens).

**Table 1:** Alpine accessions of *Dicranum acutifolium* (ac), *D. brevifolium* (br) and *D. septentrionale* (se) examined (in bold) and accessions of *D. brevifolium* and *D. septentrionale* from previous studies included in the analyses: Accession code (ISO 3166–2 country code and sequential number), morphological determination result, voucher information with herbarium location (in brackets), bedrock type, source of bedrock type information, assigned substrate condition (A acidic, N subneutral to alkaline, ? unknown), and GenBank accession numbers (*trnL–trnF*, nrITS)

Code	Morph. det	Voucher information	Bedrock	Source	Substrate	GenBank
***Dicranum acutifolium***						
**CH**	**ac**	**Switzerland, Nidwalden, Wolfenschiessen, Laubersgrat, 2355 m a.s.l., felsiger Grat, Humus in Felsspalten, 15 Okt. 2015, N. Schnyder (Z)**				**ON152900, ON152912**
**AT**	**ac**	**Österreich, Steiermark, Hochschwab mts., Trenchtling, Edelweißwiese, ca. 1750 m s.m.; alpine meadow, 20 Jun. 2006, H. Köckinger *14,984* (priv. herb. H. Köckinger)**				** − , ON152911**
***Dicranum brevifolium***						
**CH 1**	**se/br**	**Switzerland, Grisons, Tujetsch, Oberalppass, Plidutscha, 2150 m a.s.l., alpiner Rasen, Humus, 09 Jul. 2018, T. Kiebacher *1901* (Z)**	**Gneiss**	**Geological map, field observation**	**A**	** − , ON152913**
**CH 2**	**se**	**Switzerland, Valais, Obergoms, Nufenen Pass, Altstafel, tiefes Kar westlich Passhöhe, 2000 m a.s.l., auf humosem Felsabsatz, Gneis, 26 Aug. 2010, M. Lüth *6514* (Z)**	**Gneiss**	**Label**	**A**	** − , ON152914**
**CH 3**	**se**	**Switzerland, Grisons, Avers, Averstal, Fuf, Jufer Alp, 2180 m a.s.l., auf Felsblock in alpinem Rasen, 10 Sep. 2015, M. Lüth *8238* (Z)**	**Moraine of predominantely siliceous rocks**	**Geological map, field observation, associated species**	**A**	**ON152901, ON152915**
**CH 4**	**se/br**	**Switzerland, Valais, Obergoms, Gratschliecht südl. Furka Passhöhe, ca. 2490 m a.s.l., Nährstoffreicher Rasen auf Silikat, frische Erde, 24 Jul. 2012, E. Urmi *10,148* (Z)**	**Chlorit-sericitphyllite, psammitgneiss**	**Label, geological map**	**A**	**ON152902, ON152916**
		Switzerland, Valais, Ried (b. Mörel), S slope of Mt. Riederhorn, 2040 m a.s.l., open spruce forest; boulder, 14 Aug. 2004, L. Hedenäs (S)	Paragneis, Migmatit	Geological map	A	KJ651095, KJ650895
		Russia, Karachaevo-Cherkessia Prov., granitic stabilised mound, 27 Jul. 1996, Egorov (MW 9031836)	Granit	Label	A	KJ796587, HQ830342
		Russia, Tuva Prov., 12 Jun. 2007, Otnyukova (KRF)			?	KJ796588, HQ830341
		Russia, North Ossetia, Digoria, Karaugom River, right bank, 1700 m a.s.l., western slope, 20°, pine forest, 06 Sep. 2002, Korotko (MW 9031835)			?	KJ796589, HQ830343
		Russia, Yakutia, South Yakutia, 03 Aug. 2000, Kuznetzova (MW 9031839)			?	− , HQ830344
		Sweden, Hälsingland, Gnarp, Sweden. Hälsingland, Gnarp, E portion of Mt. Åsberget (near Gnarp), boulder in forest, 21 May 2010, L. Hedenäs (S B98890)	Diabas	Geological map	A	KJ651096, KJ650896
***Dicranum septentrionale***						
**AT 1**	**br**	**Austria, Vorarlberg, Lechquellengebirge: Obergschröf east of Rote Wand, ca. 2050 m a.s.l., limestone boulders between dwarf shrubs, 09 Sep. 2009, H. Köckinger *15,263* (Z)**	**Calcareous rocks**	**Label**	**N**	**ON152905, ON152919**
**AT 2**	**br**	**Austria, Steiermark, Eisenerzer Alpen: Reiting, SW-side of the summit of Gösseck, ca. 2100 m a.s.l., alpine meadow, 11 Aug. 2011, H. Köckinger *15,262* (Z)**	**Calcareous rocks**	**Field observation**	**N**	**ON152904, ON152918**
**AT 3**	**br**	**Austria, Vorarlberg, Lechtaler Alpen: Rüfikopf SE of Lech, summit, ca. 2360 m a.s.l., alpine meadow, 27 Sep. 2011, H. Köckinger *15,264* (Z)**	**Calcareous rocks**	**Field observation**	**N**	**ON152906, ON152920**
**CH 1**	**se**	**Switzerland, Grisons, Contres im Prättigau, Gaueerböden, Teilenmäder, Seebüelen, 2066 m a.s.l., 07 Aug. 2006, M. Meier *Sp34.61.01* (Z)**	**Calcareous rocks**	**Geological map**	**N**	**ON152908, ON152922**
**CH 2**	**br/ac/se**	**Switzerland, Glarus, Linthal, Obersand, unter Röti, 2150 m a.s.l., Felsbänder, Felskopf, 17 Aug. 2008, M. Meier *0808.451* (Z)**	**Calcareous rocks**	**Geological map**	**N**	**ON152907, ON152921**
**CH 3**	**se/br**	**Switzerland, Grisons, Val Müstair, Tschierv, Multetta, ca. 1840 m a.s.l., Bergföhren-Wald, kalkreicher Waldboden, 27 Jul. 2015, E. Urmi *10,453* (Z)**	**Calcareous rocks**	**Label**	**N**	**ON152909, ON152923**
**CH 4**	**br**	**Switzerland, Bern, Boltigen, Bäderhorn, Gipfel, S-Hang, 2008 m a.s.l., subalpiner Rasen mit Kalkfelsen, S-exponiert, Gesteinsrohboden, 10 Aug. 2010, H. Hofmann (Z)**	**Calcareous rocks**	**Label**	**N**	**ON152903, ON152917**
**CH 5**	**se**	**Switzerland, Vaud, Bex, N-facing slope of Mt. Haute Corde, ca. 500 m NEE of the summit, 2201 m a.s.l., alpine meadow, 25 Sep. 2017, A. Vanderpoorten, F. Collart & F. Zanatta (Z)**	**Calcareous rocks**	**Geological map**	**N**	**ON152910, ON152924**
		Austria, Alpine Rasen bei Maria Waldrast, Gemeinde Mühlbach, Tirol, 01 Aug. 1996, M. Stech B960801.2 (L)	Limestone	Geological map, field observation	N	DQ462591, KJ796539
		Finland, Alandia, Jomala, Finland. Åland, Jomala, Ytterby, W of Österviken, soil in meadow fragment, 17 Sep. 2012, L. Hedenäs & I. Bisang (S B194528)	Rich in calcium	Field observation	N	KJ796596, KJ796546
		Russia, Kamchatka, 04 Aug. 2007, Neshataeva 986 (LE)			?	KJ796585, HQ830338
		Russia, Arkhangelsk Province, 19 Jul. 2000, Churakova 864 (MW)			?	KJ796586, HQ830339
		Russia, Krasnoyarsk Territory, Putorana, 21 Jul. 1969, Kuvaev 46–8 (MW B175744)			?	− , HQ830340
		Sweden, Härjedalen, Storsjö, Sweden. Härjedalen, Storsjö, Mt. Stor-Axhögen, S-SW side, below 2nd highest peak, 1115 m a.s.l., small, wet escarpment, L. Hedenäs (S B84948)	Sandstone, partly with calcium	Geological map, field observation, associated species	N	KJ796591, KJ796541
		Sweden, Gotland, Fårö, Gotland, Fårö, Norsholmen, grazed, calcareous heath, 30 May 2006, L. Hedenäs & I. Bisang (S B74004)	Calcareous rocks	Label	N	KJ796595, KJ796545
		Sweden, Uppland, Djurö, Uppland, Djurö, Runmarö, S of Noreträsk, flat limestone rock, 07 Sep. 2002, L. Hedenäs (S B193369)	Limestone	Label	N	KJ796590, KJ796540
		Sweden, Södermanland, Nämdö, Sweden. Södermanland, Nämdö, Mörtö, Lillskogen, exposed rock, 26 Jul. 2012, L. Hedenäs (S B183369)	Limestone	Geological map	N	KJ796593, KJ796543
		Sweden, Gotland, Bunge, Sweden. Gotland, Bunge, ca. 750 m W of Bunn, forest ground, 26 May 2011, L. Hedenäs (S B162934)	Limestone	Geological map	N	KJ796592, KJ796542
		Sweden, Torne lappmark, Sweden. Torne Lappmark, Masugnsbyn, östra delen (djupa kursun), kursudal, 07 Aug 2008, T. Hallingbäck (S B194528)	Dolomite	Geological map	N	KJ796594, KJ796544

### Gene tree topology

The analysis of *trnL–trnF* sequence data resolved all species of the *D. acutifolium* complex in a supported clade (BI posteriori probability 0.99/ML bootstrap support 67; Fig. 2). They shared two synapomorphic single nucleotide polymorphisms (SNP’s) with respect to all other species considered. Within the clade, *D. brevifolium* and *D. bardunovii* lack synapomorphic mutations while *D. acutifolium* + *D. septentrionale* share two synapomorphic indels and *D. septentrionale* differs from *D. acutifolium* in two SNP’s. The phylogenetic tree of the nuclear ITS region revealed a different topology, it resolved *D. septentrionale* as rather distantly related to the other species of the complex (Fig. 3). Accessions of *D. septentrionale* were grouped in a supported clade (1/93) of a basal polytomy and differed from *D. acutifolium*, *D. brevifolium* and *D. bardunovii* in five indels and five SNP’s. The latter species appeared more closely related to eight other, morphologically well-differentiated *Dicranum*-species (e.g., *D. scoparium* Hedw., *D. leioneuron* Kindb) and were placed with these species in a supported clade (1/73). Within this clade they were grouped with weak support (0.6/-) together with *D. angustum*.

### FAMD and trait analyses

**Fig. 4:**
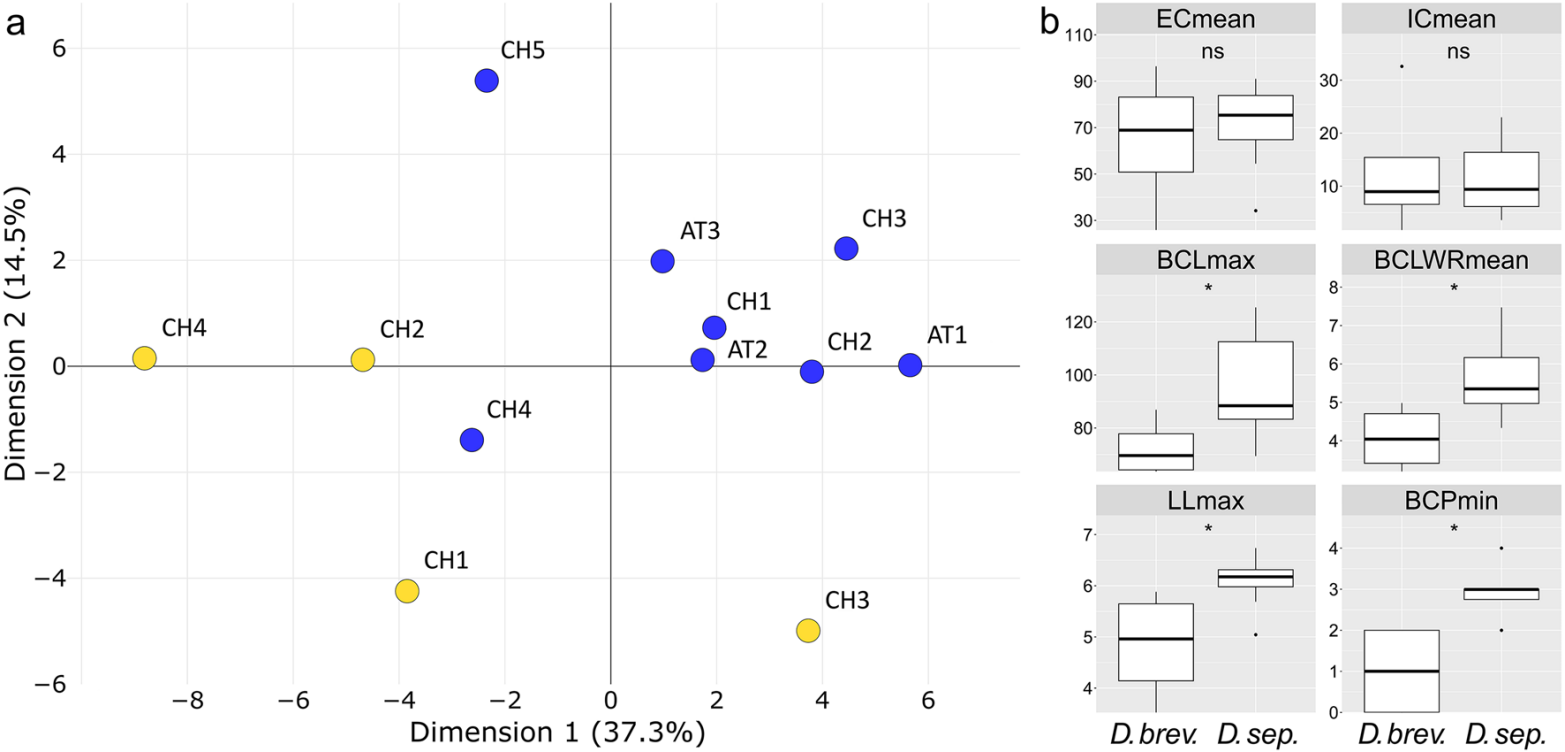
Factor analysis for mixed data **a** of 45 morphological traits of *Dicranum brevifolium* (yellow dots) and *D. septentrionale* (blue dots) and **b** differences in mean percentage of elongate (ECmean) and isodiametric cells (ICmean) in the upper lamina, maximum basal cell length (BCLmax), mean basal cell length/width ratio (BCLWRmean), maximum leaf length (LLmax) and minimum number of pores per basal cell (BCPmin). For voucher information of specimens see Table 1. Boxplots for all 45 traits are available in Fig. S7 in Supplementary Information 2

**Fig. 5:**
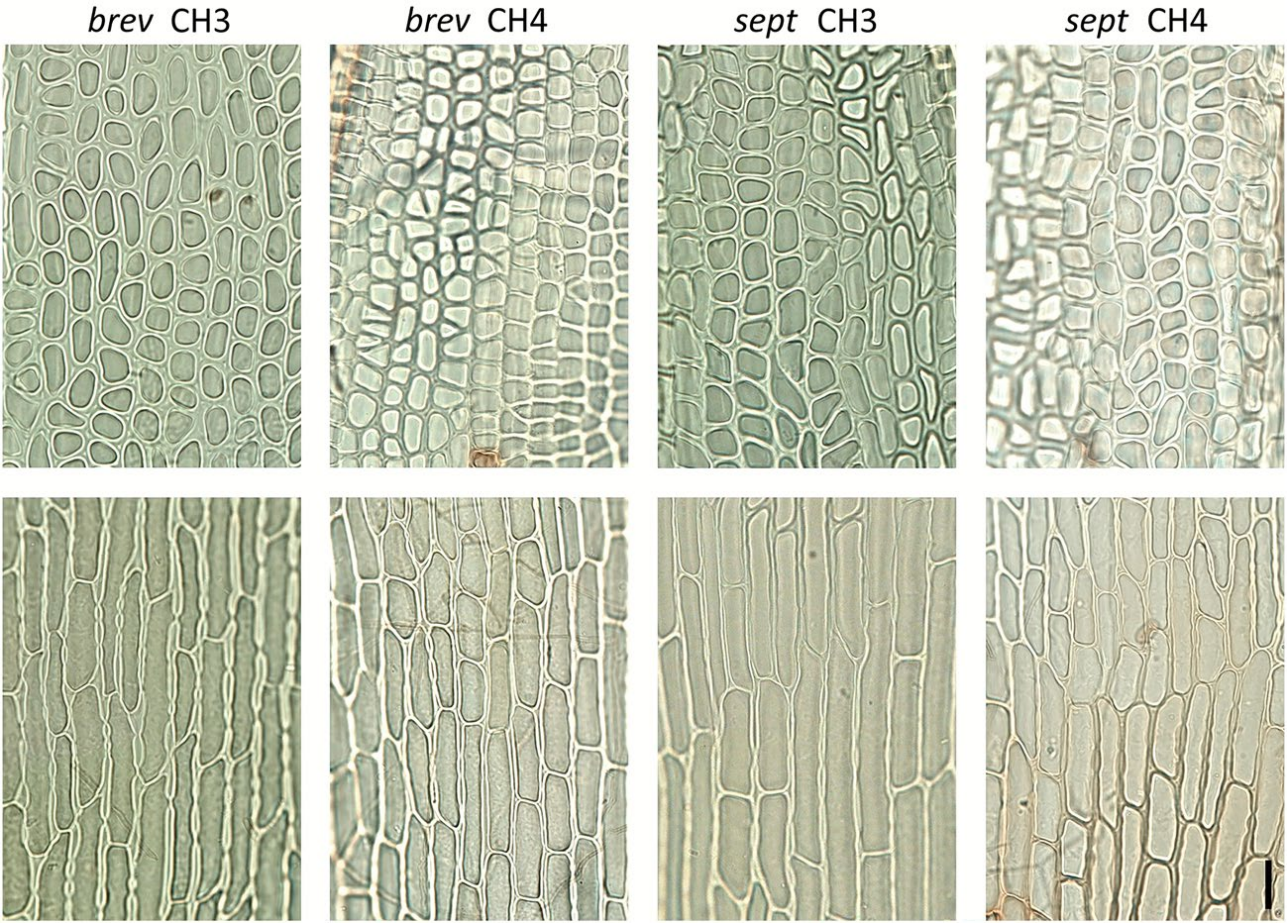
Upper and basal lamina cells of *Dicranum brevifolium* **(***brev*) and *D. septentrionale* (*sept*). The specimens CH3 of *D. brevifolium* and CH4 of *D. septentrionale* represent uncommon morphological expressions (cf. Figure 4a). For voucher information of specimens see Table 1. Scale bar 20 µm

The dimensions one and two of the FAMD analysis explained 37.3% and 14.5% of the variance respectively, revealed high within species variance and resolved the two species in two weakly separated clusters (Fig. 4a, Fig. S6 in Supplementary Information 2; a 3D plot of the first three dimensions is available in Supplementary Information 3). The analyses of individual traits failed to detect differences between the two species in the portion of elongate and isodiametric cells in the upper part of the lamina and revealed differences in leaf length, basal cell length, basal cell length/with ratio and the number of pores in basal cells, being generally larger in *D. septentrionale* (Fig. 4b, 5, Fig. S7 in Supplementary Information 2).

### Substrate reaction

All seven (out of 10) accessions of *D. brevifolium* for which substrate reaction could be classified were collected from acidic substrates, and all 16 (out of 19) accessions of *D. septentrionale* were collected from subneutral to alkaline substrates (Table 1).

## Discussion

### Evolutionary history

We found substantial discordance between plastid and nuclear sequence data regarding the phylogenetic placement of *D. septentrionale*. While the signal in *trnL–trnF* suggests the monophyly of the *D. acutifolium* complex, ITS sequences suggest that *D. septentrionale* is more deeply divergent, i.e., the most recent common ancestor of the species of the complex is shared with many other morphologically substantially different taxa. We hypothesize that the *trnL–trnF* tree better represents the evolutionary history of the species of the complex, because it coincides with their morphological similarity. Furthermore, it explains the ecological similarity of *D. septentrionale* and *D. acutifolium* to colonise subneutral to alkaline substrates by a shared evolutionary history instead of assuming an additional transition to this rare trait in the genus (Figs. 2, 3). The discordance in ITS may be due to well-known evolutionary processes, specifically gene flow and incomplete lineage sorting. To disentangle these two processes in our example would require an extended molecular dataset, but, the rare occurrence of sporophytes in the species of the *D. acutifolium* complex, as well as their longevity suggest that incomplete lineage sorting is a more likely explanation (Copetti et al. 2017; Meleshko et al. 2018). For instance, a recent genome-wide study of long-lived and rather rarely sexually reproducing peatmosses detected incomplete lineage sorting as the main source of discordance between nuclear and organellar genomes (Meleshko et al. 2021). On the other hand, gene flow seems to predominate in short-lived species that frequently produce sporophytes (Košnar et al. 2012; Linde et al. 2020; McDaniel et al. 2010; Nieto-Lugilde et al. 2018).

Within the *D. acutifolium* complex, the *trnL–trnF* data suggest a sister position of *D. acutifolium* and *D. septentrionale* and not of *D. brevifolium* and *D. septentrionale,* although the latter two are morphologically very similar whereas *D. acutifolium* can usually be easily distinguished from them. Consequently, the similarity of *D. brevifolium* and *D. septentrionale* would represent an example of either stasis or convergence (Struck et al. 2018). As outlined above, the sister position of *D. septentrionale* and *D. acutifolium* is supported by their substrate specificity. Both occur on subneutral to alkaline substrates whereas *D. brevifolium* and *D. bardunovii* occur on acidic substrates. Since the preference for acidic substrates is a conservative trait in the genus and transitions to alkaline substrates are rare (Figs. 2, 3), the ability of *D. septentrionale* and *D. acutifolium* to colonise alkaline substrates probably originated from the same evolutionary event and is most likely unrelated to the discordance in ITS sequence data of *D. septentrionale*, because *D. acutifolium* does not share the discordance.

Interestingly, the genetic distances between morphologically poorly differentiated species such as *D. brevifolium* and *D. septentrionale* are similar or even larger as between morphologically well-differentiated species (e.g., *D. elongatum* Schleich. ex Schwägr. and *D. fragilifolium* Lindb. in *trnL–trnF*; *D. leioneuron* and *D. scoparium* in ITS). Overall, most other *Dicranum* species considered are morphologically well-differentiated. All but four (*D. bardunovii, D. dispersum, D. ignatovii, D. septentrionale*) were described more than 100 years ago and accepted in most taxonomic concepts since their description (e.g., Hill et al. 2006; Hodgetts et al. 2020; Ireland 1971; Limpricht 1886; Mönkemeyer 1927; Nyholm 1954; Suzuki 2016), hence, the genetic distance does not necessarily reflect the morphological distance. Possibly, in the absence of morphological differentiation, a morphological optimum for a particular habitat has already been reached and is selectively maintained, whereas adaptive radiation is still possible at the physiological level. Moreover, it is likely that (near-) cryptic taxa can also be detected in other *Dicranum* species if a larger number of specimens are studied along their ecological gradients.

### Substrate specificity and cryptic species

*Dicranum septentrionale* occurs on subneutral to alkaline substrates, whereas its near-cryptic counterpart *D. brevifolium* occurs on acidic substrates. The two species thus represent ecological vicariants. Cryptic or near-cryptic species associated with ecological vicariance have been observed in various groups of plants, animals and fungi (e.g., Boissin et al. 2011; Douglas et al. 2011; Douhan et al. 2008; Reis et al. 2020) but were rarely reported from bryophytes (Kiebacher et al. 2022). Only recently, Kiebacher et al. (2022) described a similar example in the epilithic moss *Lewinskya killiasii* (Müll. Hal.) Kiebacher, Köckinger & Jan Kučera (Orthotrichaceae). The two subspecies of this taxon occur on siliceous and carbonate rocks, respectively. Divergent substrate preference is probably a common phenomenon among (near-)cryptic bryophytes but this has not been exhaustively demonstrated, possibly because many examples are still unknown and hidden in species considered insensitive to substrate reaction. In contrast, cryptic speciation has often been shown to correlate with biogeographical patterns (e.g., Bakalin et al. 2020; Hedenäs 2017; Hutsemékers et al. 2012; Shaw 2001). Due to the long evolutionary history of many bryophyte species (Laenen et al. 2014), presumably both processes in concert often contributed to the cryptic diversity observed today (Hedenäs 2017; Hedenäs et al. 2020). In the case of *D. brevifolium* and *D. septentrionale,* however, a clear biogeographical pattern could not be observed. Both taxa occur throughout Russia (Tubanova et al. 2010), Scandinavia (Lang et al. 2014) and the Alps and the small overlap of the distribution areas observed in Russia (Tubanova et al. 2010) can now be explained by the different geology of these regions. The assessment of the ecological differentiation of *D. brevifolium* and *D. septentrionale* was largely limited to the Alps and Scandinavia (Table 1), because it was difficult to recover reliable substrate information of individual specimens from Russia. However, Tubanova et al. (2010) noted that in the Kola Peninsula *D. brevifolium* is much more common than *D. septentrionale,* whereas the latter is more common in the Kamchatka Peninsula, and this coincides with the geology of the two regions. The Kola Peninsula is dominated by acidic bedrock types (Mitrofanov et al. 1995) and the Kamchatka peninsula by alkaline basalts rich in magnesia (Portnyagin et al. 2015). Thus, this provides partial support for our observations in the Alps and Scandinavia.

### Occurrence and morphological differentiation in the Alps

Our data confirm the presence of three species of the *Dicranum acutifolium* complex, *D. acutifolium*, *D. brevifolium* and *D. septentrionale*, in the Swiss and Austrian Alps. *Dicranum septentrionale* is probably the most frequent species of the complex in the region, whereas *D. brevifolium* hitherto is confirmed only from Central Switzerland. Our search for recent collections of *D. brevifolium* s.l. revealed only a few specimens from siliceous bedrock, suggesting that *D. brevifolium* is rare and that it should be prioritised in conservation. The same may apply to Scandinavia and Europe in general because *D. brevifolium* s.l. is normally given as basiphilous (Hedenäs and Bisang 2004; Nyholm 1987). *Dicranum acutifolium* seemingly occurs scattered in the limestone ranges of both Austria and Switzerland (Köckinger et al. 2021, Köckinger in litt). Additional research is required to examine the distribution of these species at a more detailed geographical resolution.

*Dicranum acutifolium* is mostly recognizable by its narrow and hardly-crisped leaves in dry condition and the elongated, variably shaped cells in the median and upper part of the lamina (Hedenäs and Bisang 2004; Tubanova et al. 2010). In contrast, *D. brevifolium* and *D. septentrionale* are characterised by strongly-crisped leaves in dry condition (Fig. 1c) and the median and upper cells rather uniformly shaped (oblate, quadrate or short rectangular). On the other hand, our data indicate that the morphological distinction of *D. brevifolium* and *D. septentrionale* in the Alps is not straightforward. This is especially true for the proportion of elongate and isodiametric upper lamina cells, which was proposed as a critical difference (Tubanova et al. 2010) based on specimens from Russia. This trait was similar in Alpine specimens of the two species (Figs. 4b, 5) and is possibly strongly influenced by environmental conditions. For example, the predominantly elongate upper lamina cells in the *D. brevifolium* collection from Avers (Lüth 8238; Table 1; CH3 in Fig. 5; Supplementary Information 1) could be due to the unusually humid microhabitat, partly shaded by vascular plants. In part, the analyses of individual traits also confirmed differences observed in specimens from Russia. *Dicranum brevifolium*, on average, has shorter basal cells, a smaller length/with ratio of basal cells and fewer pores per basal cell, and our data additionally suggest that its leaves are shorter (Tubanova et al. 2010; Fig. 4, Fig. S7 in Supplementary Information 2; Tubanova et al. 2010). However, due to overlaps in essentially all traits and the low number of specimens at hand for the analyses, these differences should be used with caution to distinguish the species and we recommend verifying morphological determinations molecularly, especially if the substrate is in conflict with the morphological diagnosis (e.g., *D. brevifolium* on calcareous bedrock). In such cases, it is more reliable to label specimens as *D. brevifolium* s.l.

The occurrence of (near-)cryptic species that are ecologically and genetically differentiated indicates that morphological concepts should not uncritically be interpreted to reflect the species diversity of bryophytes. Because (near-)cryptic species can have similar genetic differentiation as morphologically well differentiated ones, and may occupy different niches, they should not be ignored in conservation perspectives. To uncover hidden diversity in bryophytes it seems worthwhile to focus on species with presumably low substrate specificity.

### Supplementary Information

Below is the link to the electronic supplementary material.

Supplementary file1 (XLSX 20 KB)

Supplementary file2 (PDF 3255 KB)

Supplementary file3 (HTML 3782 KB)

## Data Availability

Sequence data are available in GenBank (https://www.ncbi.nlm.nih.gov/genbank), the alignments are available at the Zenodo repository (https://zenodo.org; 10.5281/zenodo.7768294) and voucher specimens are deposited in Z + ZT.
