# Supplementary material for: Morphological, genetic and ecological divergence in near-cryptic bryophyte species widespread in the Holarctic: the Dicranum acutifolium complex (Dicranales) revisited in the Alps
Source: J Plant Res. 2024 Mar 23;137(4):561–74. doi: 10.1007/s10265-024-01534-3 (PMC11230997; doi:10.1007/s10265-024-01534-3)
Supplement: Supplementary file 2 — Supplementary file2 (PDF 3255 KB) [file 10265_2024_1534_MOESM2_ESM.pdf]

Journal of Plant Research

Morphological, genetic and ecological divergence in near-cryptic bryophyte species widespread in the Holarctic: The *Dicranum acutifolium* complex (Dicranales) revisited in the Alps

Thomas Kiebacher[1, 2*] & Péter Szövényi[2,3]

[1] Stuttgart State Museum of Natural History, Rosenstein 1, D-70191 Stuttgart, Germany

[2] Department of Systematic and Evolutionary Botany, University of Zurich UZH, Zollikerstrasse 107, CH-8008 Zurich, Switzerland

[3] Zurich-Basel Plant Science Center (PSC), ETH Zürich, Tannenstrasse 1, 8092 Zürich, Switzerland

* Corresponding author, E-mail: thomas.kiebacher@smns-bw.de

Supplementary Information 2

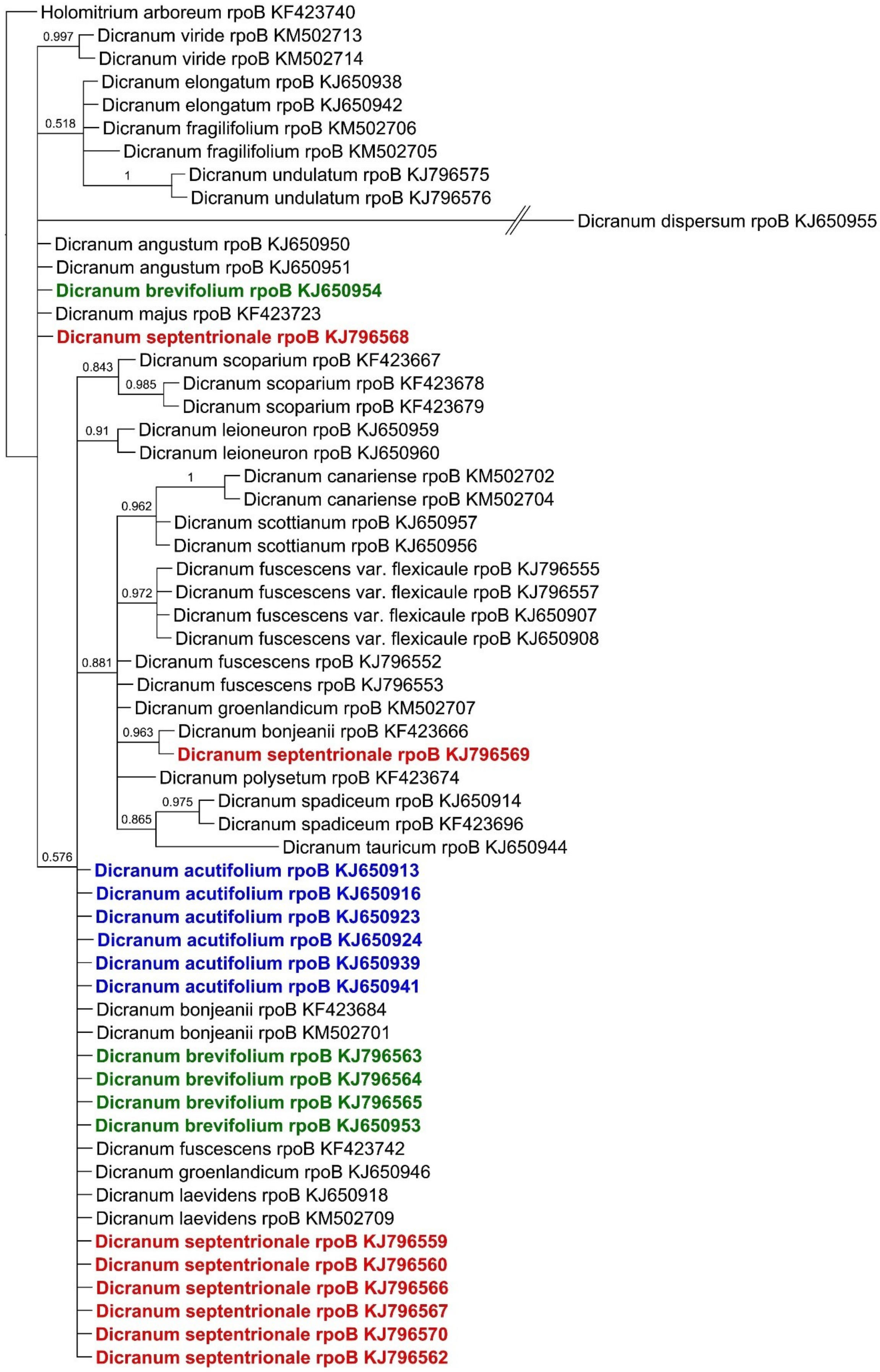

Fig. S1 Bayesian inference from plastid *rpoB* sequence data based on the dataset of Lang et al. (2015). Numbers above branches are Bayesian posterior probabilities ≥ 0.5

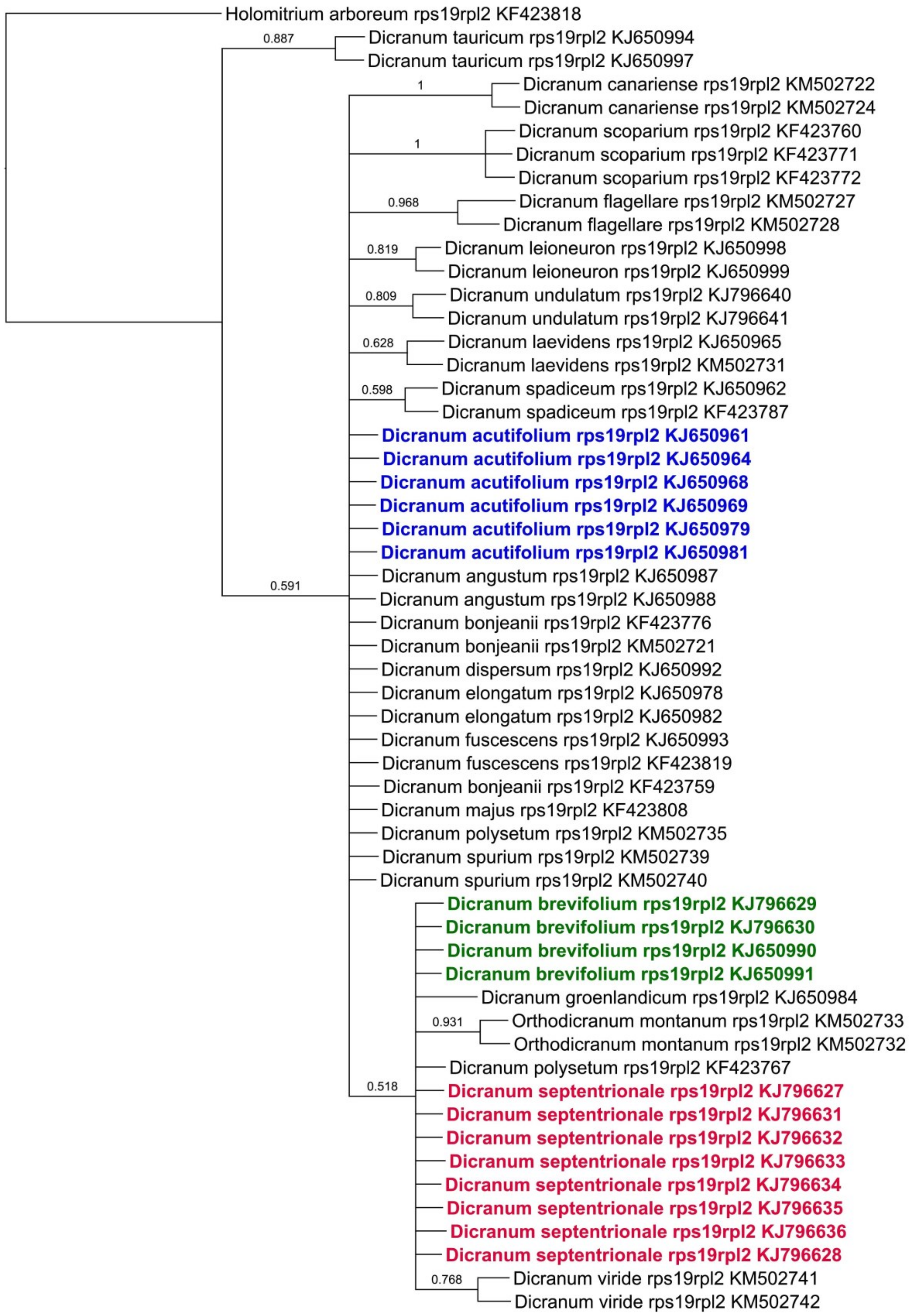

Fig. S2 Bayesian inference from plastid *rps19–rpl2* sequence data based on the dataset of Lang et al. (2015). Numbers above branches are Bayesian posterior probabilities ≥ 0.5

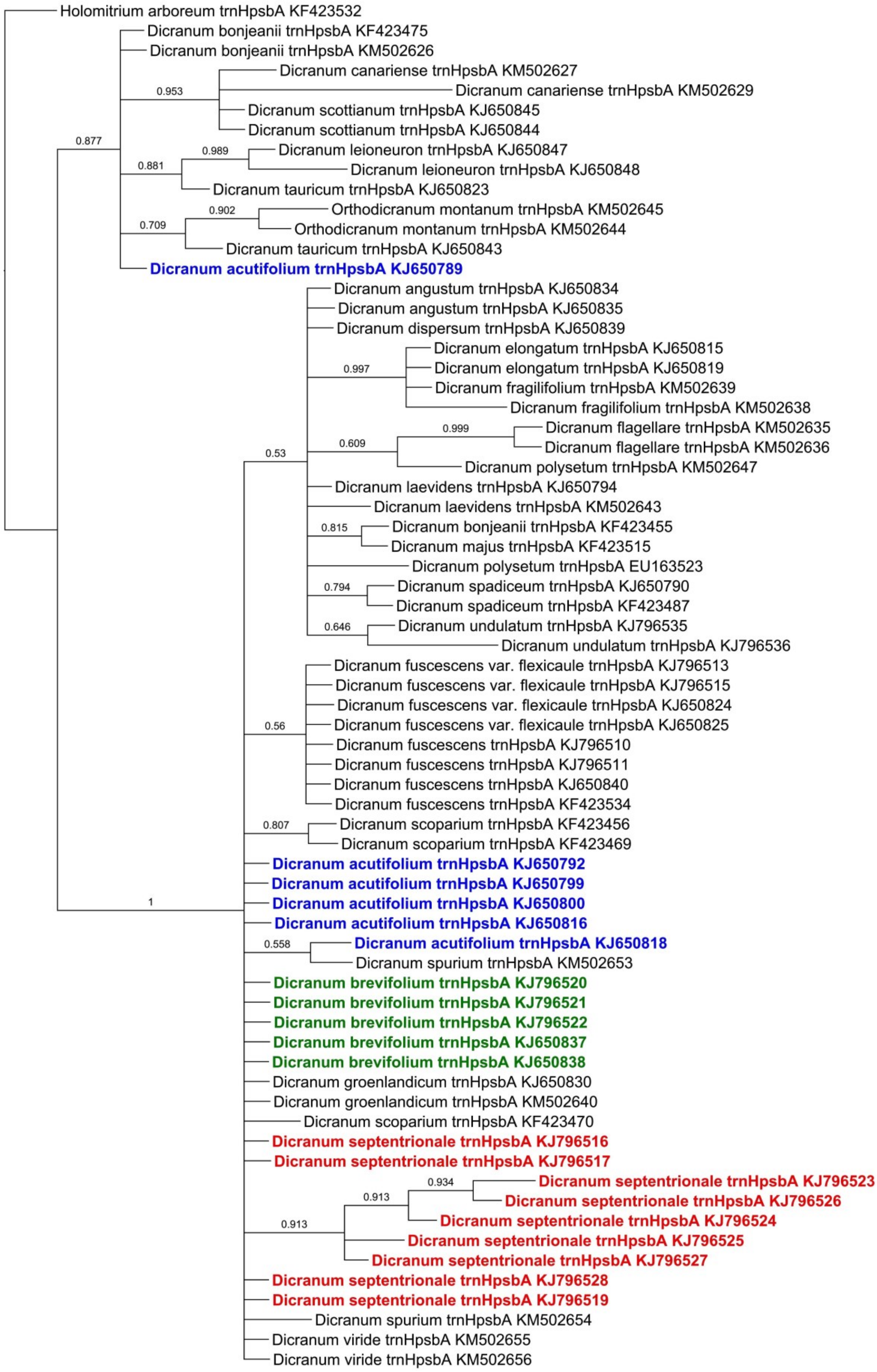

Fig. S3 Bayesian inference from plastid *psbA–trnH* sequence data based on the dataset of Lang et al. (2015). Numbers above branches are Bayesian posterior probabilities ≥ 0.5

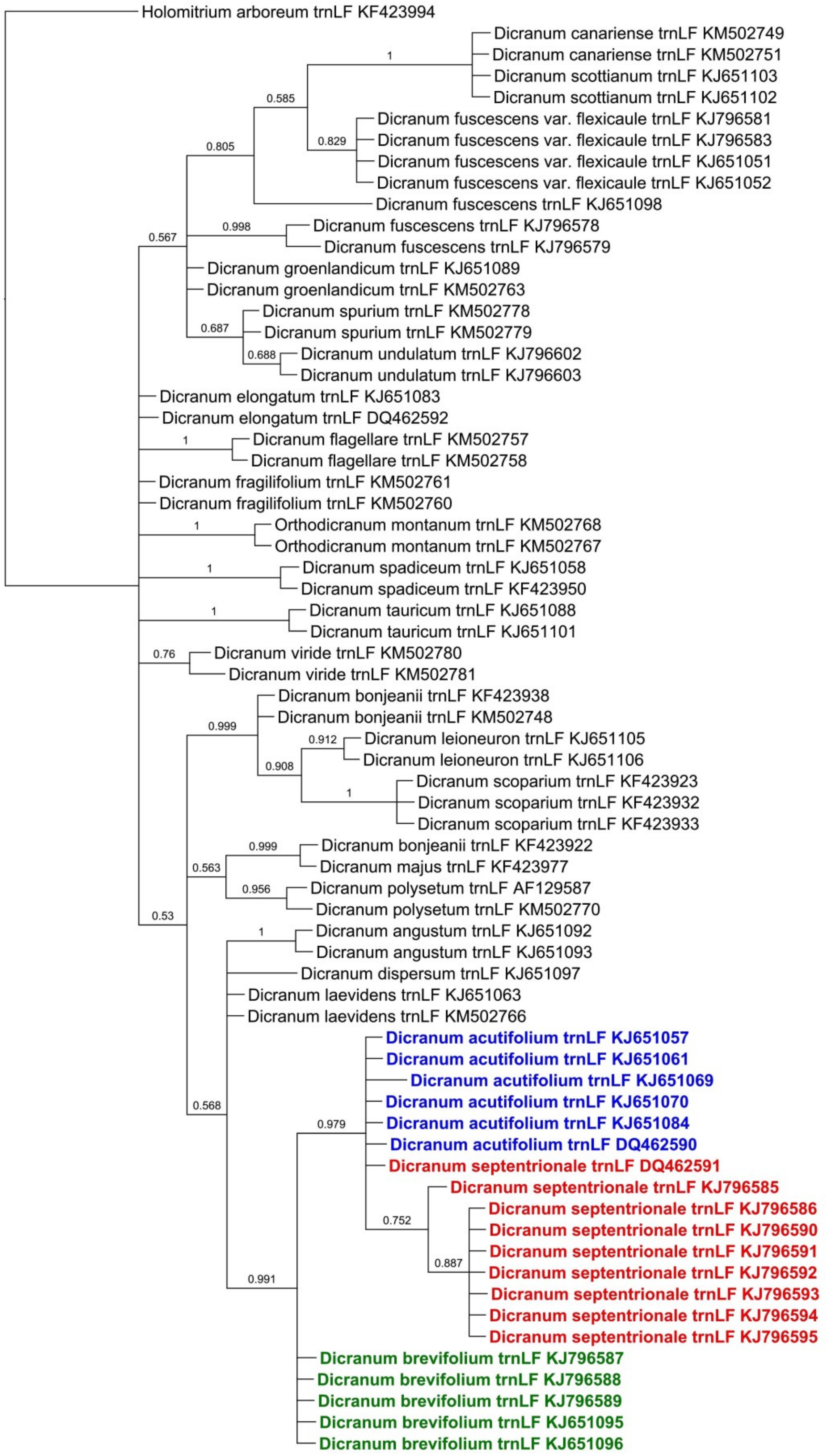

Fig. S4 Bayesian inference from plastid *trnL–trnF* sequence data based on the dataset of Lang et al. (2015). Numbers above branches are Bayesian posterior probabilities ≥ 0.5

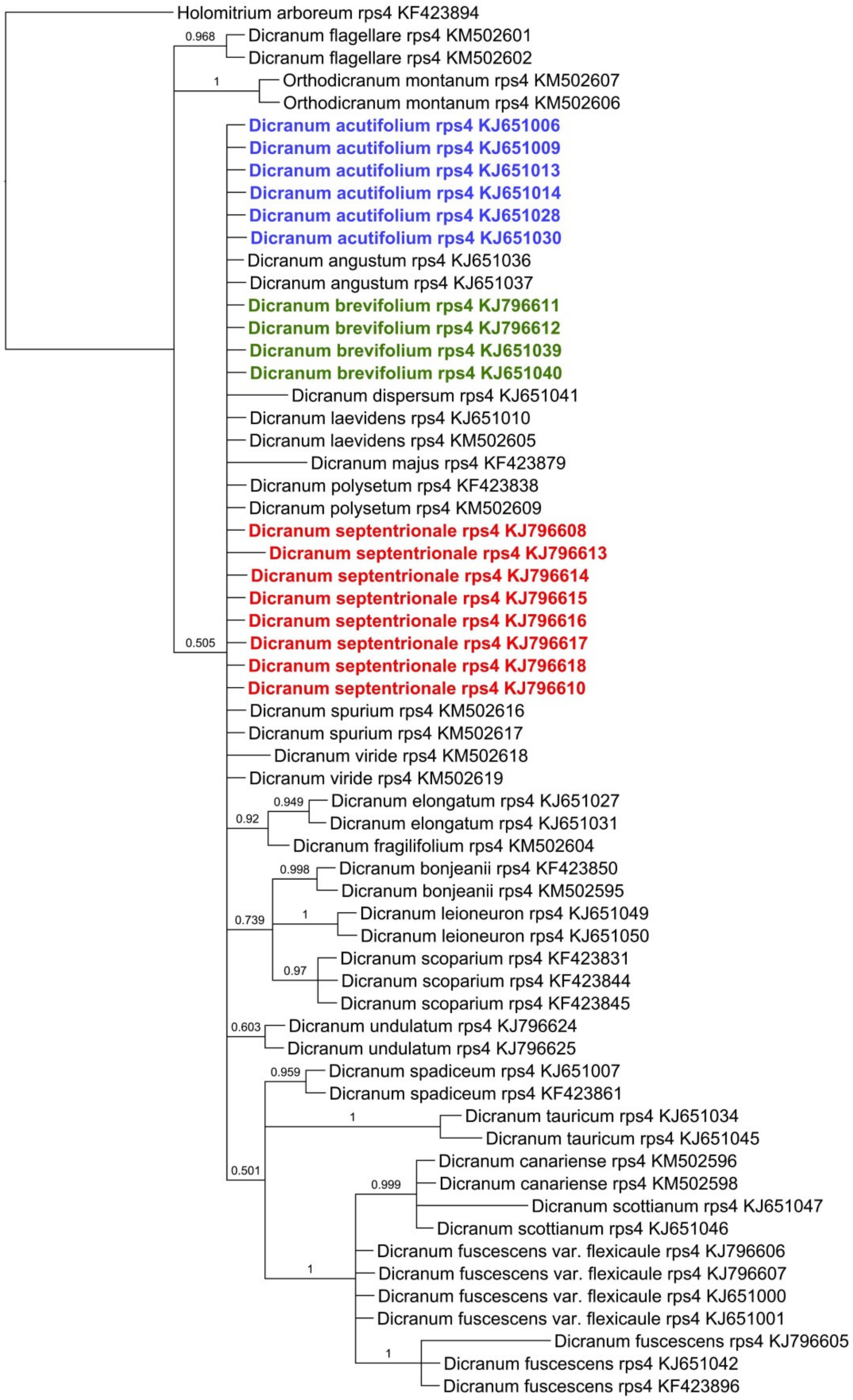

Fig. S5 Bayesian inference from plastid *trnT–rps4* sequence data based on the dataset of Lang et al. (2015). Numbers above branches are Bayesian posterior probabilities ≥ 0.5

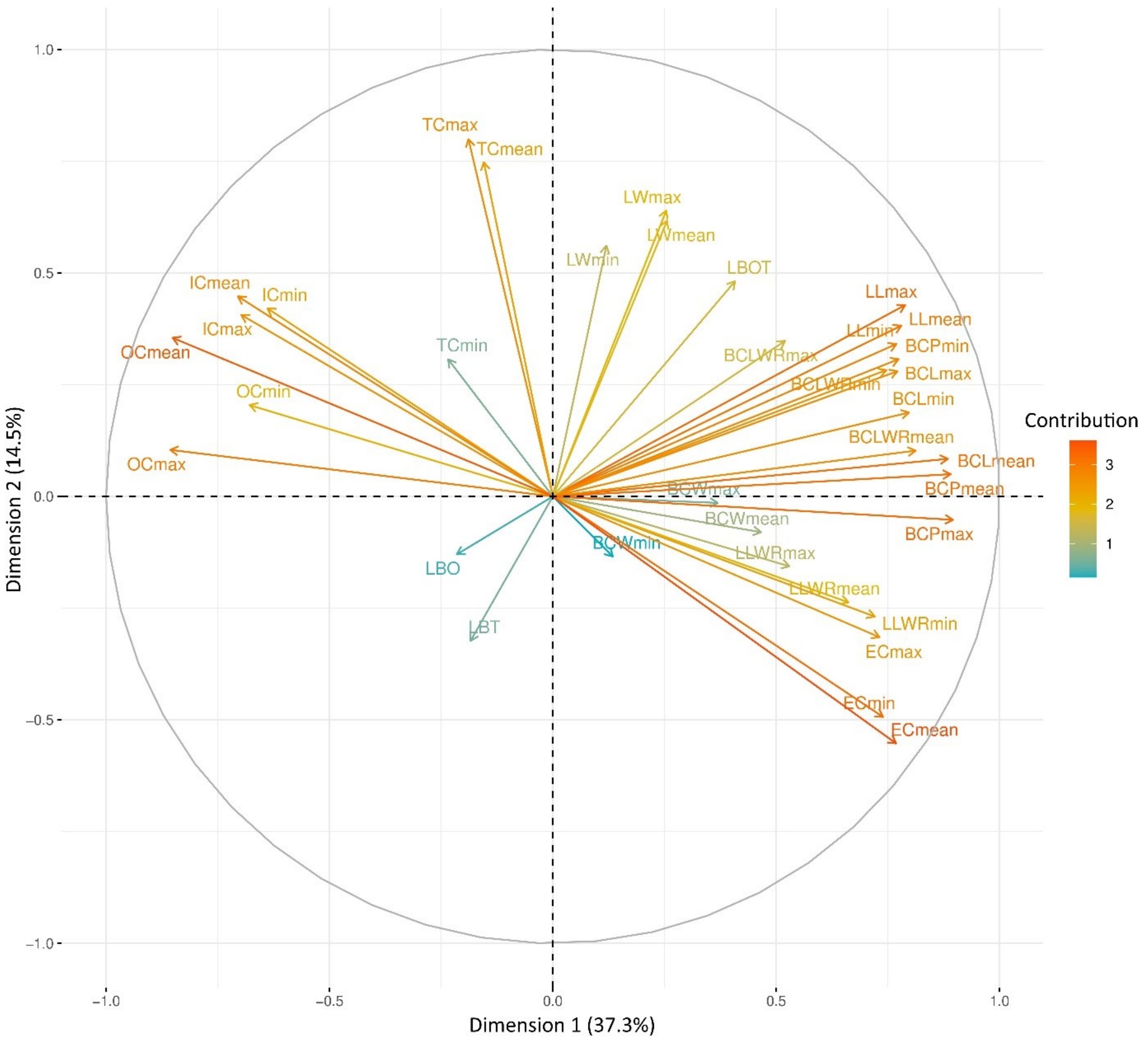

Fig. S6 Contribution of 45 morphological traits to the first two dimensions of the factor analysis for mixed data. Minimum (min), maximum (max), mean and median of leaf length and width (LL, LW), leaf length/width ratio (LLWR), dorsal costa ornamentation in upper ¼ (CO), leaf margin ornamentation in upper ¼ (LM), basal cell length and width (BCL, BCW), basal cell length/width ratio (BCLWR), number of pores of basal cells (BCP), percentage of elongate (EC), isodiametric (IC), oblate (OC) and triangular (TC) cells in upper 1/2 – 2/3 of the lamina and number of leaves with ovate (LBO), ovate-triangular (LBOT) and triangular leaf base (LBT)

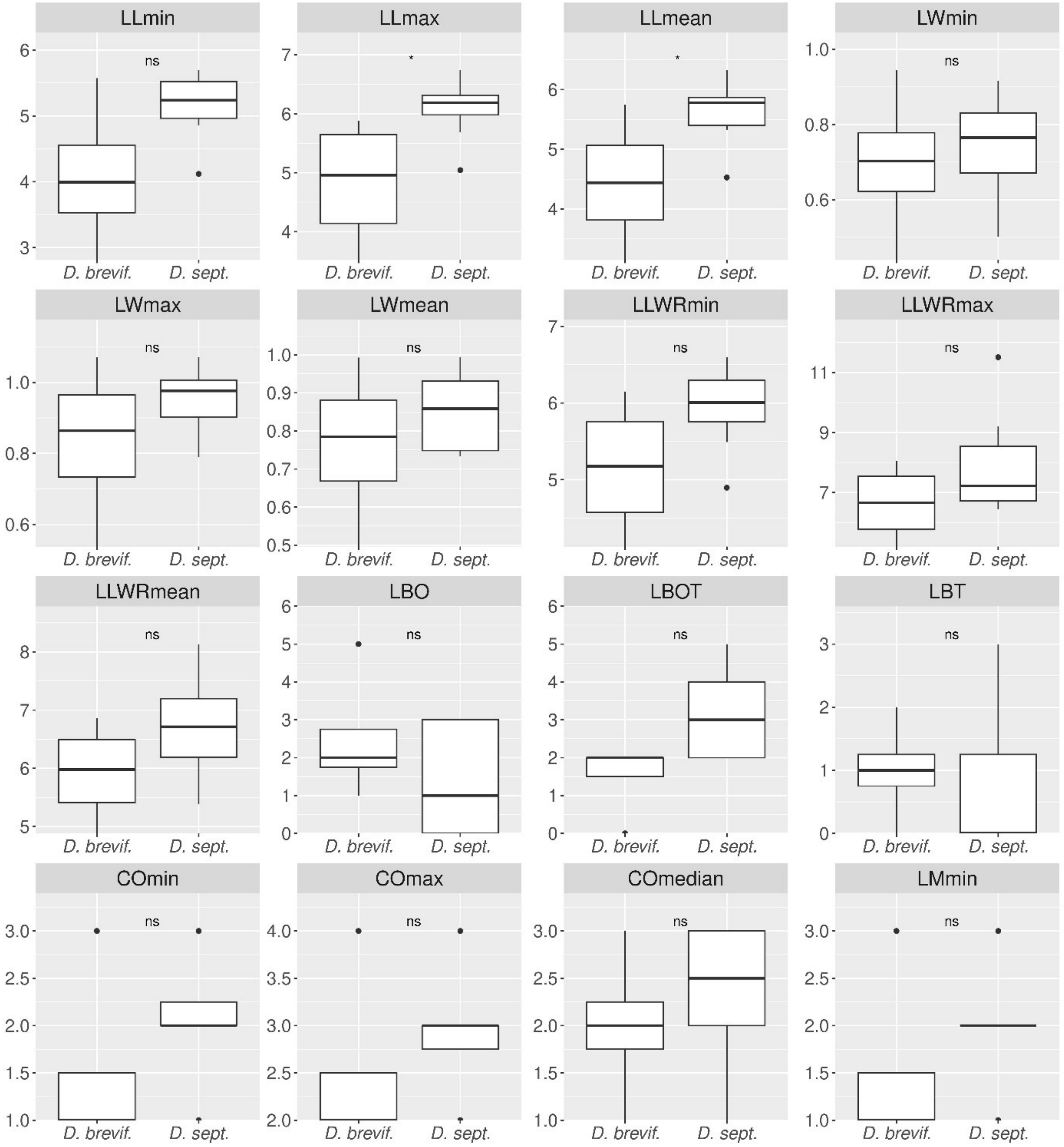

Fig. S7 Morphological differences between *Dicranum brevifolium* and *D. septentrionale* in 45 traits. Significance level according to Wilcoxon signed-rank test: * $p < 0.05$, ns $p \geq 0.05$. Minimum (min), maximum (max), mean and median of leaf length and width (LL, LW; mm), leaf length/width ratio (LLWR), dorsal costa ornamentation in upper ¼ (CO; 1 smooth, 2 mammillose, 3 denticulate, 4 dentate), leaf margin ornamentation in upper ¼ (LM; 1 smooth, 2 denticulate, 3 dentate 4 spinosely dentate), basal cell length and width (BCL, BCW; µm), basal cell length/width ratio (BCLWR), number of pores of basal cells (BCP), percentage of elongate (EC), isodiametric (IC), oblate (OC) and triangular (TC) cells in upper 1/2 – 2/3 of the lamina and number of leaves with ovate (LBO), ovate-triangular (LBOT) and triangular leaf base (LBT)

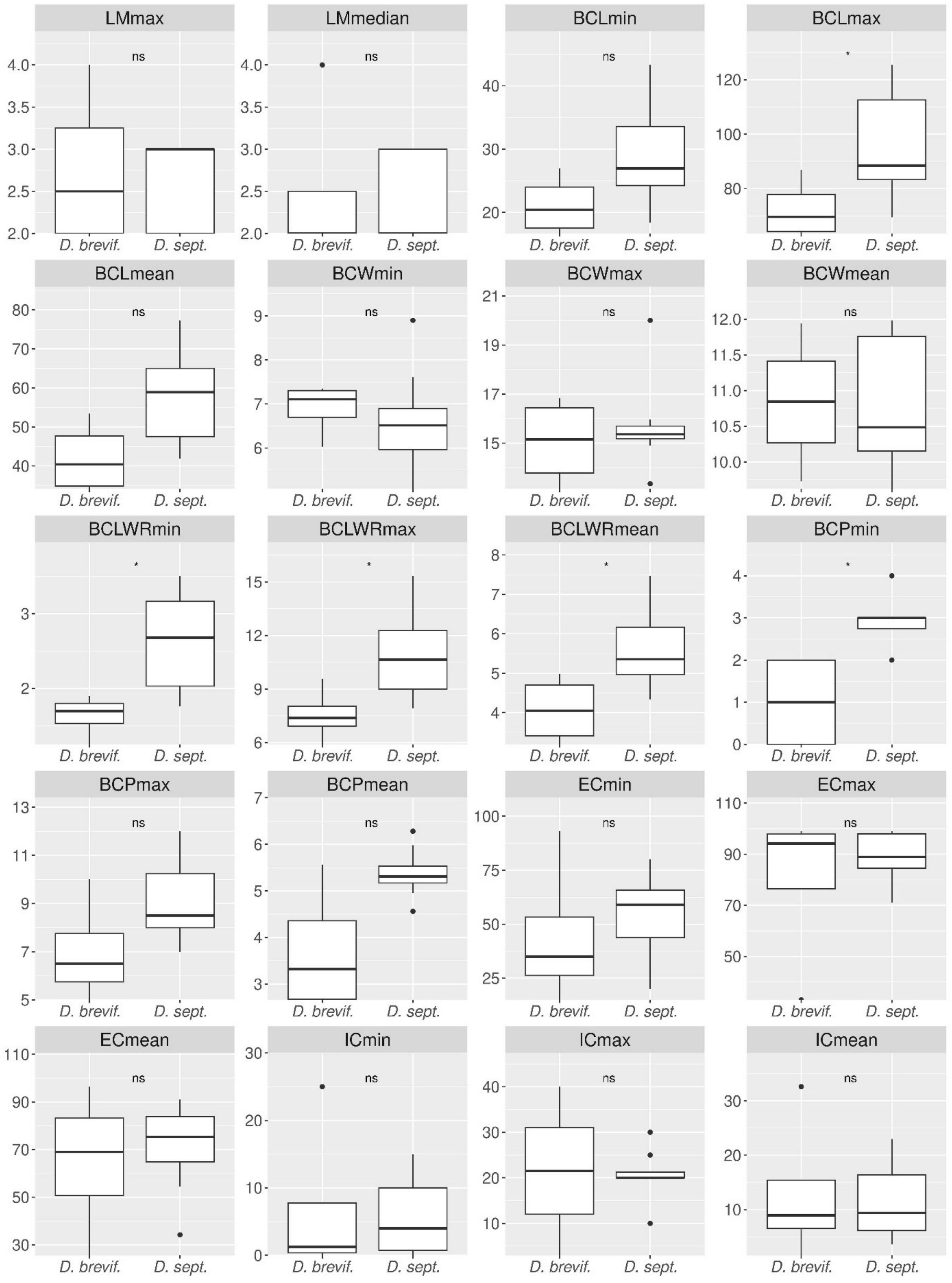

Fig. S6 continued

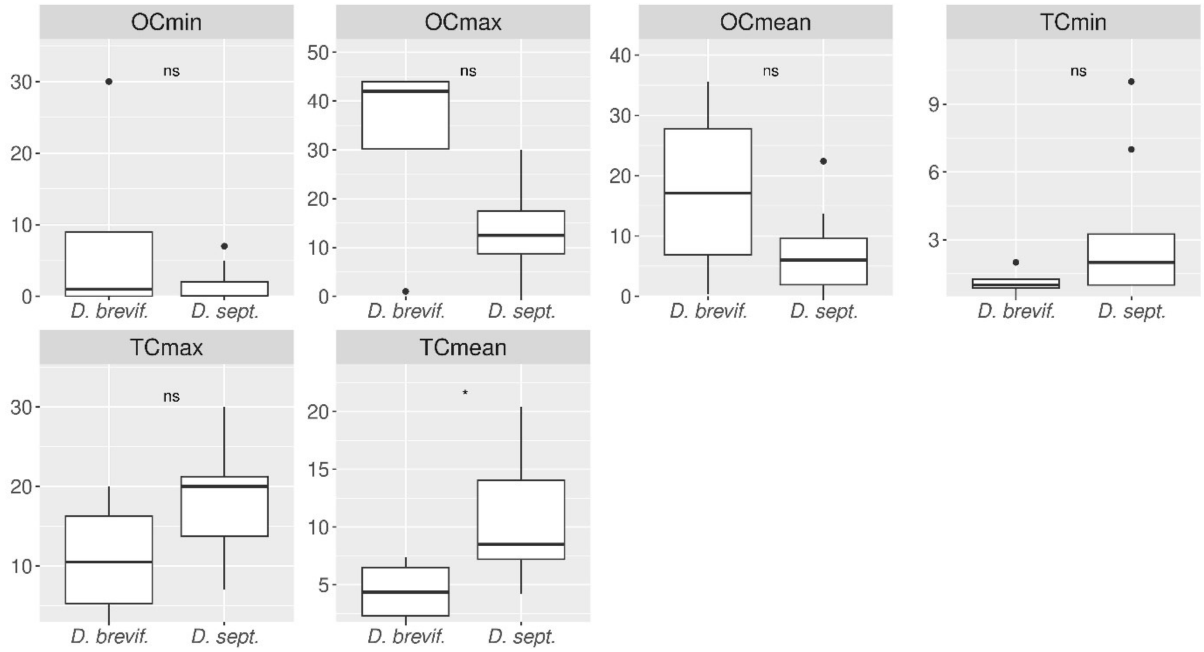

Fig. S6 continued
